# Jack of All Trades, Master of All: A Positive Association between Habitat Niche Breadth and Foraging Performance in Pit-Building Antlion Larvae

**DOI:** 10.1371/journal.pone.0033506

**Published:** 2012-03-15

**Authors:** Erez David Barkae, Inon Scharf, Zvika Abramsky, Ofer Ovadia

**Affiliations:** 1 Department of Life Sciences, Ben-Gurion University of the Negev, Beer-Sheva, Israel; 2 Institute of Zoology, Johannes Gutenberg University of Mainz, Mainz, Germany; University of Debrecen, Hungary

## Abstract

Species utilizing a wide range of resources are intuitively expected to be less efficient in exploiting each resource type compared to species which have developed an optimal phenotype for utilizing only one or a few resources. We report here the results of an empirical study whose aim was to test for a negative association between habitat niche breadth and foraging performance. As a model system to address this question, we used two highly abundant species of pit-building antlions varying in their habitat niche breadth: the habitat generalist *Myrmeleon hyalinus*, which inhabits a variety of soil types but occurs mainly in sandy soils, and the habitat specialist *Cueta lineosa*, which is restricted to light soils such as loess. Both species were able to discriminate between the two soils, with each showing a distinct and higher preference to the soil type providing higher prey capture success and characterizing its primary habitat-of-origin. As expected, only small differences in the foraging performances of the habitat generalist were evident between the two soils, while the performance of the habitat specialist was markedly reduced in the alternative sandy soil. Remarkably, in both soil types, the habitat generalist constructed pits and responded to prey faster than the habitat specialist, at least under the temperature range of this study. Furthermore, prey capture success of the habitat generalist was higher than that of the habitat specialist irrespective of the soil type or prey ant species encountered, implying a positive association between habitat niche-breadth and foraging performance. Alternatively, *C. lineosa* specialization to light soils does not necessarily confer upon its superiority in utilizing such habitats. We thus suggest that habitat specialization in *C. lineosa* is either an evolutionary dead-end, or, more likely, that this species' superiority in light soils can only be evident when considering additional niche axes.

## Introduction

Habitat utilization spectrum is an important dimension of the ecological niche. A broadly accepted explanation for the variation in niche breadth among closely related species along such central niche axes, is the existence of a trade-off between the ability of a species to utilize a wide range of resources and its performance when exploiting only one or a few of them [1]–[6]. In other words, if adaptation to an additional habitat entails a fitness loss in the former, species having a narrow spectrum of habitat utilization (i.e., habitat specialists) should perform better than those utilizing a wider range of habitats (i.e., habitat generalists), but only within a narrower habitat spectrum. Habitat generalists, on the other hand, can inhabit more habitats, but they never achieve the performance of the habitat specialist on any one of them. Empirical studies, however, have not always been able to confirm this trade-off in performance associated with niche breadth (e.g. [7]–[10]), suggesting that this principle is less trivial and common than initially assumed.

Trap-building predators, such as web-building spiders or pit-building antlions, are opportunistic predators which depend heavily on their physical environments [11]–[13]. Pit-building antlion species can greatly differ in their habitat niche breadth and preferred habitats. Although antlions often prefer inhabiting shaded habitats, they may also reside in open habitats exposed to direct sun [13]–[15]. In addition, antlions exhibit extensive variation in their preferences for soil/sand particle sizes ([16], [17] see also [18] for a comparison between antlions and wormlions). Despite their preferences for different soil types, however, antlions will sometimes construct pits in less desirable habitats, but because such pits are usually smaller, they can cause reductions in prey capture rate [12], [19].

We report here on the results of an empirical study whose aim was to test for a negative association between habitat niche breadth and foraging performance. As a model system we used two highly abundant species of pit-building antlions that vary in their habitat utilization spectrum: the habitat generalist *Myrmeleon hyalinus* inhabits a variety of soil types but occurs mainly in sandy soils, and the habitat specialist *Cueta lineosa* is restricted to light soils (finer textured soils) such as loess [14]. The two antlions are similarly sized and have comparable life cycles. We hypothesized that the habitat specialist, *C. lineosa*, would construct pits and respond to prey faster than the habitat generalist, *M. hyalinus*, in the loess soil, resulting in higher prey capture success. We also hypothesized that the habitat specialist's superiority in its preferred habitat of light soils would be significantly reduced in the sand compared to the habitat generalist, whose average performance should not vary between the soils.

## Methods

### Study species and habitats-of-origin

We collected *M. hyalinus* larvae under different tamarisk trees located in Nahal Secher (N 31°06′, E 34°49′), a sandy area 15 km south of the city of Beer-Sheva, Israel, and brought them to the laboratory. *M. hyalinus* is the most abundant pit-building antlion in Israel [14]. The larvae attain maximal lengths of about 10 mm and body masses of up to 0.06 g before pupating [20]. They inhabit a variety of soil types but occur mainly in sandy soils [14]. In addition, we collected *C. lineosa* larvae from the loessial plains near Beer-Sheva (N 31°16′, E 34°50′). Occurring mainly in the Israeli Negev desert, *C. lineosa* also exists in several small populations located in central and northern Israel, but is restricted to light soils, such as loess [14]. The two antlions are similarly sized and have comparable life cycles. Although they largely overlap in their geographical distribution, they rarely overlap in their microhabitat use. Specifically, *M. hyalinus* prefers shaded microhabitats [14], [21], while *C. lineosa* is mainly found in open microhabitats exposed to direct sunlight [14]. Therefore, it is unlikely that interference competition exists between the two antlion species. However, it is possible that they indirectly compete for their arthropod prey (i.e., exploitation competition). All required permits and approvals for this work were obtained from Israel's Nature and National Parks Protection Authority, permit no. 2010/37830. In compliance with all the relevant laws and regulations prevailing in Israel, self-regulation and accountability of local programs by an Institutional Animal Care and Use Committee (IACUC) are not applicable for the use of invertebrates in research (Israel's Animal Welfare Act 1984).

### Experimental design & statistical analysis

The study comprised of three complementary experiments: 1) Foraging behavior experiment, investigating the foraging behavior of both species in two different soil types, loess and sand, while using only one prey ant species. 2) To test if prey capture success is sensitive to prey species, we repeated the first experiment using three different prey ant species collected from the two field sites mentioned earlier. 3) Habitat selection experiment, testing whether the two species are capable of distinguishing between the two soils and choosing the soil type providing a higher prey capture success.

Prior to all experiments, antlions were fed with one flour beetle larva (mean larva mass ∼1 mg), starved for 10 days in small plastic cups (diameter of 4.5 cm, filled with about 3 cm of sand or loess), and then weighed using an analytical scale (CP224S, Sartorius AG, Goettingen, Germany; accurate to 0.1 mg). Our previous experience with antlion larvae indicates that this procedure is useful for standardizing their hunger level and physiological state before they enter the experiment [22].

#### Foraging behavior experiment

**Figure 1:**
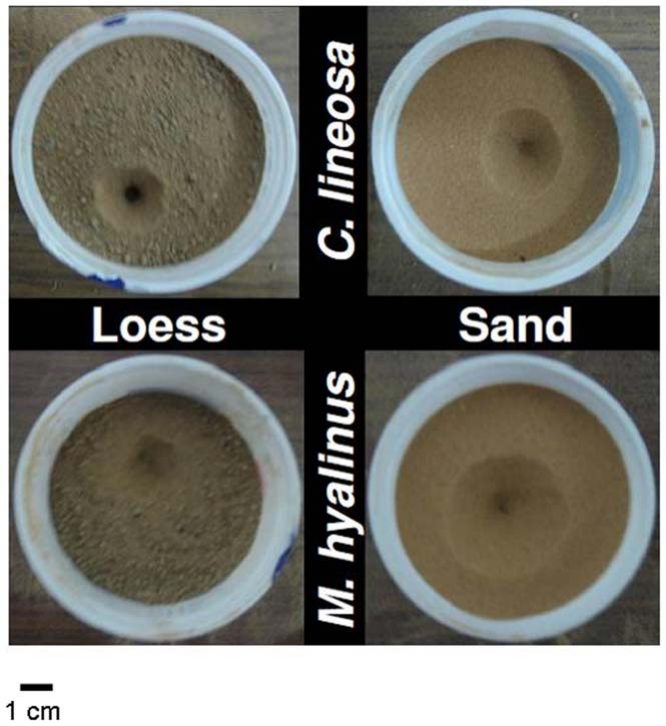
Experimental design showing pit construction of both antlion species in the two soil types.

Sixty individuals of each species were divided into two groups characterized by similar body size distributions (Kolmogorov-Smirnov two sample test, P = 0.20 and P = 0.52 for *C. lineosa* and *M. hyalinus*, respectively). Body size distribution also did not differ between species (Kolmogorov-Smirnov two sample test, P = 0.38). To avoid competition and potential cannibalism [23], [24], we introduced single larvae into round plastic cups (diameter = 8.5 cm, depth = 6 cm) filled either with sand from Nahal Secher or with loess brought from the loessial plains near Beer-Sheva (i.e., soil type treatment, Fig. 1). All larvae were kept in the same room under an identical night/day photoperiod (12∶12 h), temperature of 27.8°C and 70% r.h. Among the sand grains, 7.97% were larger than 0.25 mm, 78.65% were between 0.125–0.25 mm, 11.54% were between 0.062–0.125 mm, and 1.84% were smaller than 0.062 mm [25]. The smaller loess particles comprised 2.8% grains larger than 0.2 mm, 32.4% grains between 0.05–0.2 mm, 48.8% grains between 0.05–0.002 mm, and 16% grains smaller than 0.002 mm [26].

Immediately after placing the larvae in the cups, we monitored the foraging behavior of the two species in both soils. Specifically, we documented the time to soil diving by measuring the time from placing the larvae on the soil surface to a complete disappearance of the larvae under the soil, time required to construct a pit measured as the time from initial movement of the larvae, until pit was completed and no further sand tossing was observed, and pit diameter and depth using a caliper (accuracy of 0.1 mm). Pit diameter was calculated as the average of two successive measurements of the diameter at the soil surface, while pit depth was measured from the soil surface to the bottom of the antlion pit (similarly to [22], [27]) We also provided antlions with ants [*Messor aegyptiacus*; mean ant mass = 1.6 mg±0.2 mg (±1 S.D; N = 20)] and documented their response times to prey (similar to [28]) and their prey capture success. This ant species inhabits the loess plains of the Negev desert and the Arava valley, but does not occur in the sand dunes of the western Negev desert [29]. Each antlion received only one prey item, as the foraging behavior of antlions varies between fed and non-fed larvae [22] and between experienced and inexperienced larvae [28]. With the exception of prey capture success, all response variables (i.e., pit diameter, pit depth, time to soil diving, time to pit construction, and response time to prey) were analyzed using two-way ANCOVAs, with species and soil as the explanatory variables and body mass as the covariate. Our prey capture success analysis included only those individuals that responded to prey. Since the proportion of individual *C. lineosa* responding to prey was very low in the sand, we had to provide ants to a larger number of individuals (i.e., 90), to ensure that our analysis would be more balanced. Differences in prey capture success were tested using a logistic regression [30]. All data were log transformed prior to the analysis. Finally, since individuals were randomly placed in plastic cups located in the same room (i.e., same conditions) and because observations on randomly selected individuals took place in the same day, there was no need to include block or time effects in the analysis.

#### Prey capture success experiment

There is a substantial variation in morphological and behavioral characteristics among prey ant species, such as thickness of cuticle [31], mandible properties [32], body size and running speed [33], behavioral defense mechanisms [34] and habitat use [35]. Such differences can be also reflected in the probability of being captured by antlion larvae. Thus, we carried out a second experiment whose aim was to test if the prey capture success of these antlions is sensitive to their prey ant species. Specifically, we collected 180 new larvae of each species from the field. Similarly to the foraging behavior experiment, larvae were individually stocked into plastic cups, identical to those described in the foraging behavior experiment, and were randomly assigned to one of the two soil type treatments (Fig. 1). In addition, we collected three different species of ants from the field: *M. aegyptiacus* [mean ant mass = 1.6 mg±0.2 mg (±1 S.D; N = 20)] mainly occurring in the loess plains of the Negev desert and the Arava valley, but absent from the sand dunes of the western Negev desert [29]; *Pheidole pallidula* [mean ant mass = 0.3 mg±0.1 mg (±1 S.D; N = 23)] and *Messor ebeninus* [mean ant mass = 4 mg±0.3 mg (±1 S.D; N = 22)]. These two latter ant species are distributed all over Israel while inhabiting both loess and sandy soil habitats [35]. Antlion larvae were divided into three groups, each provided with a different ant species as prey (i.e., prey species treatment). As in the foraging behavior experiment, differences in prey capture success were tested using a logistic regression [30].

#### Habitat selection experiment

To test if antlions are capable of distinguishing between the two soils, we collected 60 new larvae of each species from the field. We used 25×17 cm aluminum trays partitioned into two halves of equal sizes. Using cardboard as a barrier, we filled the trays with sand and loess at opposite halves, and then removed the cardboard. We placed a single antlion larva in the middle of the aluminum tray, and recorded the location of the antlion pit after 72 h (i.e., sand or loess), as a previous study indicated that a 2-day period is sufficient for pit construction [21], and that this pattern does not vary with time [36]. Trays were kept under identical conditions as in the previous experiments. We tested antlions habitat selection using a χ^2^ test of independence. All statistical analyses were performed using SYSTAT v. 11 (SYSTAT Software, San Jose, CA, USA).

## Results

### Foraging behavior experiment

We could not detect significant differences in the proportion of pits constructed between species or soil types (*M. hyalinus* sand: 87%, *M. hyalinus* loess: 83%, *C. lineosa* loess: 87%, *C. lineosa* sand: 90%; χ^2^ = 0.0001, df = 1, P = 0.997).

**Figure 2:**
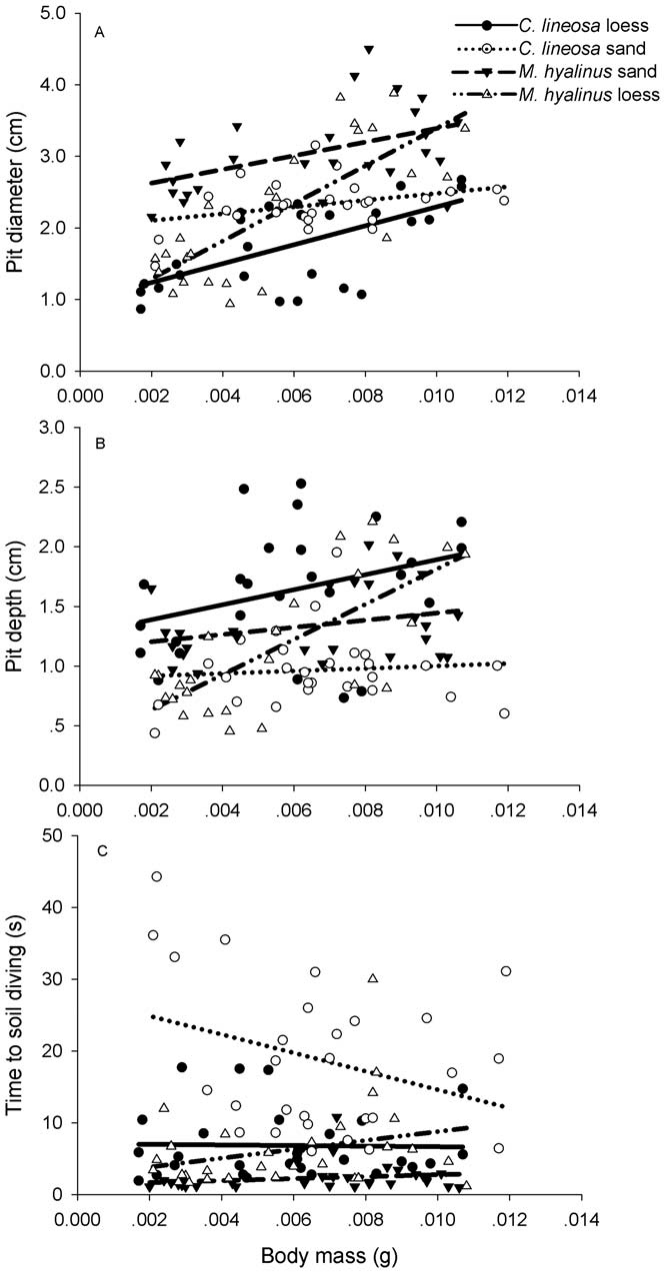
Pit diameter (A), pit depth (B), and time to soil diving (C) of both antlion species in the two soils as functions of their body masses. Both antlion species constructed pits with larger diameters in the sand than in the loess. *M. hyalinus* pits were larger than those of *C. lineosa*, irrespective of soil type (R^2^ = 0.625). Loess pits of *C. lineosa* were deeper than those of *M. hyalinus*, but the sand pits of the latter were deeper than those of the former (R^2^ = 0.436). *M. hyalinus* dives into the soil faster than *C. lineosa*, but this pattern is evident only in the sand (R^2^ = 0.612).

There was an overall significant increase in pit diameter with body mass (F_1,96_ = 52.682, P<0.001), and this pattern was consistent between species (species×body mass interaction; F_1,96_ = 1.783, P = 0.209), but not among soil types (soil×body mass interaction; F_1,96_ = 11.964, P<0.001). This latter two-way interaction was caused by the faster increase in pit diameter, evident in both species, in the loess (Fig. 2A). The three-way soil×species×body mass interaction was not significant (F_1,96_ = 1.677, P = 0.198). In both species, pit diameter was larger in the sand than in the loess (F_1,96_ = 7.991, P = 0.006). Additionally, *M. hyalinus* pits were larger than those of *C. lineosa* irrespective of soil type (saturated GLM: F_1,96_ = 3.514, P = 0.063; reduced GLM including all three main effects and the significant soil×body mass interaction: F_1,99_ = 29.72, P<0.001; Fig. 2A).

Pit depth increased significantly with body mass (F_1,96_ = 20.59, P<0.001) and differed between soil types (deeper in general in the loess; F_1,96_ = 5.01, P = 0.028; Fig. 2B). Notably, the three-way species×soil×body mass interaction was significant (F_1,96_ = 3.86, P = 0.052), indicating that the increase in pit depth with body mass was not consistent between species and soil types. *C. lineosa* pits in loess were deeper than their pits in sand over the entire range of body masses examined. The depths of pits dug by *M. hyalinus* increased with body mass at a faster rate in the loess than in the sand (Fig. 2B). As a result, pits of larvae weighing <6.9 mg were deeper in the sand while those of larvae weighing >6.9 mg were deeper in the loess (Fig. 2B). *C. lineosa* dug significantly deeper loess pits than those of *M. hyalinus* (F_1,48_ = 13.48, P<0.001), but the sand pits of *M. hyalinus* were deeper than those of *C. lineosa* (F_1,50_ = 26.49, P<0.001; Fig. 2B).

The relationship between time to soil diving and body mass was not consistent between species and soil types (species×soil×body mass interaction: F_1,111_ = 72.01, P<0.001). Specifically, time to soil diving in *M. hyalinus* did not change significantly with body mass in either soil (r = 0.337, P = 0.068 and r = 0.227, P = 0.228 for sand and loess, respectively), but it was shorter in the sand than in the loess across the entire range of body masses examined (Fig. 2C). In contrast, *C. lineosa* larvae, again in a pattern that was consistent over the entire range of masses, dived faster in the loess than in the sand. Moreover, time to soil diving in this species decreased significantly with body mass in the sand but not in the loess (r = −0.375, P = 0.049 and r = 0.062, P = 0.749 for sand and loess, respectively; Fig. 2C). No significant differences in time to soil diving in the loess were evident between the two species (F_1,55_ = 0.43, P = 0.516); however, in the sand, *M. hyalinus* dived significantly faster than *C. lineosa* (F_1,55_ = 198.58, P<0.001; Fig. 2C).

**Figure 3:**
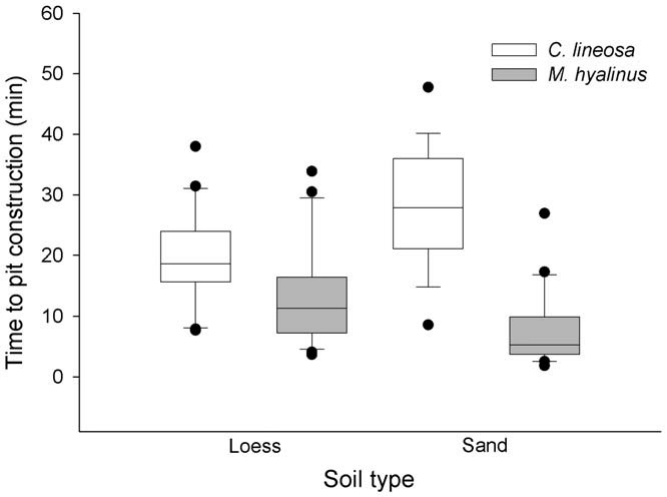
Time to pit construction of antlions. *M. hyalinus* larvae constructed pits faster than *C. lineosa* larvae, irrespective of the soil type. Key: median (horizontal lines in boxes), inter-quartile range (boxes), 95th and 5th percentiles (vertical bars), outliers (black dots).

Time to pit construction did not vary significantly with body mass (F_1,76_ = 0.02, P = 0.882) or between soil types (F_1,76_ = 1.21, P = 0.275). *M. hyalinus* constructed pits at a faster rate than *C. lineosa* (F_1,76_ = 67.54, P<0.001; Fig. 3), a pattern that was much stronger in sand (species×soil interaction, F_1,76_ = 16.20, P<0.001; Fig. 3) than in loess.

**Figure 4:**
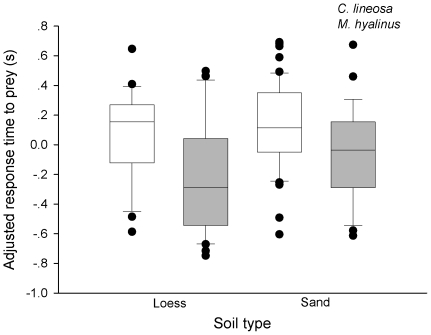
Response time of antlions to prey. *M. hyalinus* larvae responded to prey faster than *C. lineosa* larvae, irrespective of the soil type. Key: median (horizontal lines in boxes), inter-quartile range (boxes), 95th and 5th percentiles (vertical bars), outliers (black dots).

There was a significant negative correlation between response time to prey and body mass (F_1,125_ = 4.3068, P = 0.04) that was consistent between species (species×body mass interaction; F_1,125_ = 0.0008, P = 0.978). To control for the effect of body mass, we analyzed the residuals obtained by regressing response times against body masses. Between soil types, we could not detect significant differences in response time to prey (F_1,126_ = 0.95, P = 0.33). However, we found that the response of *M. hyalinus* to prey was significantly faster than that of *C. lineosa* (F_1,126_ = 18.79, P<0.0001; Fig. 4), and this pattern was consistent between soil types (soil×species interaction; F_1,126_ = 3.4, P = 0.06; Fig. 4).

**Figure 5:**
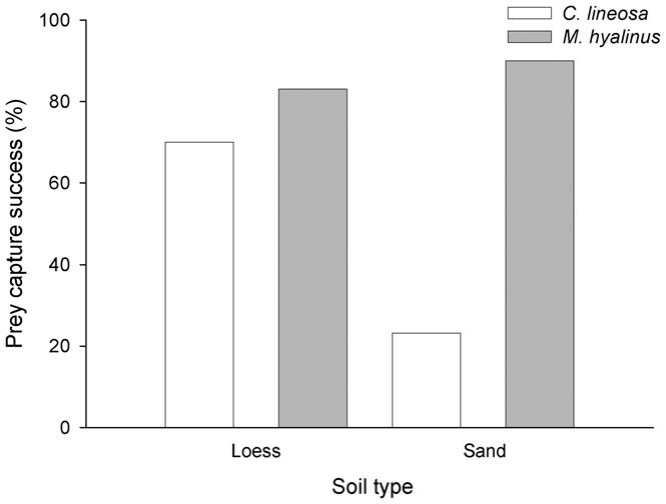
Prey capture success of antlions in the foraging behavior experiment. Prey capture success of *M. hyalinus* varied little between soils, but that of *C. lineosa* was markedly reduced when put in the sand. Notably, the prey capture success of the habitat generalist *M. hyalinus* was higher than that of the habitat specialist *C. lineosa* in both soil types.

Using a logistic regression, we found that the effect of soil type on prey capture success was not consistent between the two antlions (species×soil type interaction; Table 1). Specifically, in *M. hyalinus* prey capture success was relatively high in both soil types (90% and 83% in the sand and loess, respectively; Fig. 5). In contrast, *C. lineosa* prey capture success was significantly lower in the sand than in the loess (23% and 70%, respectively; Fig. 5). Notably, the prey capture success of *M. hyalinus* was higher than that of *C. lineosa* in both soil types (Fig. 5).

**Table 1:** Logistic regression analysis examining prey capture success in the foraging behavior experiment.

Parameter	D.F	Wald statistics	p-value
Species	1	23.46	<0.001
Soil type	1	4.632	0.031
Antlion mass	1	0.847	0.357
Species×Soil type	1	11.574	<0.001
Intercept	1	3.067	0.080

Prey capture success differed significantly between the two antlion species and soil types. However, the corresponding interaction term was also significant, implying that observed differences in prey capture success between antlion species were not consistent among soil types (see text for more detail).

### Prey capture success experiment

**Figure 6:**
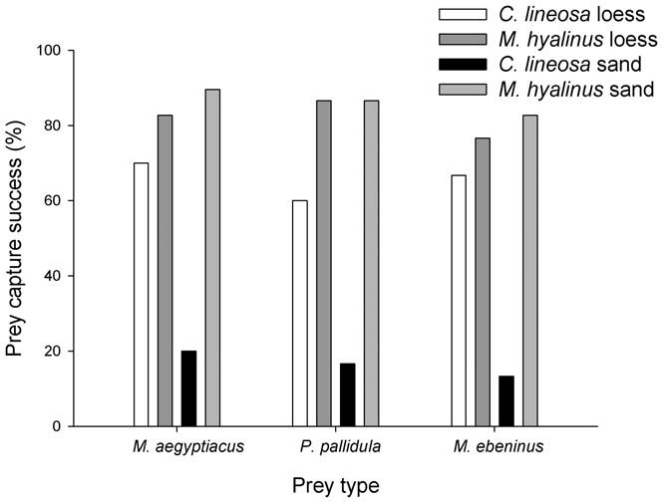
Prey capture success of antlions encountering different prey ant species. Prey capture success of both species did not vary among the different ant prey species. Prey capture success of *M. hyalinus* varied little between soils, but that of *C. lineosa* was markedly reduced when put in the sand. Notably, the prey capture success of the habitat generalist *M. hyalinus* was higher than that of the habitat specialist *C. lineosa* in both soil types.

Using a logistic regression, we found that prey capture success of the two antlions did not vary significantly among prey ant species (Table 2). Similarly to the results obtained in the foraging behavior experiment, we found that the effect of soil type on prey capture success was not consistent between the two antlions (species×soil type interaction; Table 2). Specifically, prey capture success of *M. hyalinus* did not vary significantly between soil types and was higher than that of *C. lineosa* irrespective of the prey ant species (Fig. 6). However, in *C. lineosa* prey capture success dropped by ∼50% when switching from the loess to the sandy soil and this pattern was consistent among prey ant species (Fig. 6). Note that also when we examined only the loess data, prey capture success of *M. hyalinus* was significantly higher than that of *C. lineosa* (P = 0.011 after applying a Bonferroni correction for multiple testing).

**Table 2:** Logistic regression analysis testing for differences in prey capture success of antlions encountering different prey ant species.

Parameter	D.F	Wald statistics	p-value
Antlion species	1	60.231	<0.001
Soil type	1	15.215	<0.001
Prey species	2	1.703	0.427
Antlion mass	1	3.271	0.071
Antlion species×Soil type	1	19.312	<0.001
Prey species×Soil type	2	0.246	0.884
Antlion species×Prey species	2	1.427	0.490
Antlion species×Soil type×Prey species	2	0.592	0.744
Intercept	1	13.258	0.021

Antlion prey capture success was not affected by the prey ant species they encountered, but it differed significantly between antlion species and soil types. Again, there was a significant Antlion species×Soil type interaction, implying that differences in prey capture success between antlions were not consistent among soil types (see text for more detail).

### Habitat selection choice experiment

**Figure 7:**
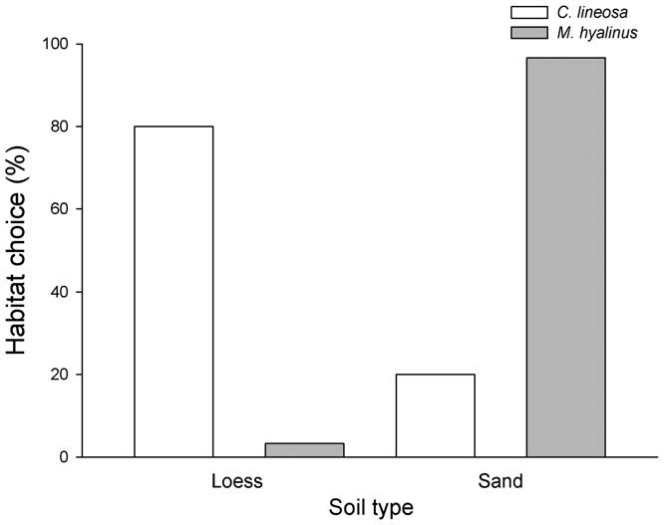
Habitat choice of antlions. Both antlion species discriminate between soils, choosing the soil type providing a higher prey capture success and characterizing their primary habitat-of-origin.

Using a choice experiment we found that ∼97% of *M. hyalinus* larvae preferred constructing pits in the sand (χ2 = 52.27, df = 1, P<0.0001). *C. lineosa* larvae, on the other hand, preferred to construct their pits in the loess (∼80%; χ^2^ = 21.60, df = 1, P<0.0001; Fig. 7).

## Discussion

We used two highly abundant species of pit-building antlions, varying in their habitat niche breadth, to test the classical assumption that adaptation to an additional habitat entails a fitness loss in the former one (e.g. [6], [37]). We show that both antlions are capable of discriminating between the two soils, with each showing higher preference to the soil type providing higher prey capture success and characterizing its primary habitat-of-origin. As expected, only small differences in the foraging performances of the habitat generalist, *M. hyalinus*, were evident between the two soils, while the performance of the habitat specialist, *C. lineosa*, was markedly reduced in the alternative sandy soil (Fig. 6). In both species, pit diameter was larger in the sand than in the loess. *M. hyalinus* pits were larger than those of *C. lineosa* irrespective of soil type. Although loess pits of *C. lineosa* were deeper than those of *M. hyalinus*, the sand pits of the latter were deeper than those of the former. *M. hyalinus* dived into the soil faster than *C. lineosa*, but this pattern was evident only in the sand. Remarkably, in both soil types, the habitat generalist *M. hyalinus* constructed pits and responded to prey faster than the habitat specialist *C. lineosa*. As a result, the former enjoyed higher prey capture success, implying a positive association between habitat niche-breadth and foraging performance. Furthermore, this pattern was not sensitive to the prey ant species encountered. These findings clearly indicate that the habitat specialization of *C. lineosa* to light soils (e.g., loess) does not necessarily confer upon this species superiority in utilizing such habitats, at least under the temperature range of this study.

Remarkably, the widely accepted theoretical assumption, suggesting that adaptation to an additional habitat should confer inferiority in utilizing the former (see references in [38]), has been empirically demonstrated in some studies (e.g. [39]–[42]). For example, Laverty & Plowright [39], showed that a flower specialist bumblebee, *Bombus consobrinus*, is more effective in foraging on its specialized flower, specifically in handling time and ability to find nectar, compared with two closely related flower generalists, *Bombus fervidus* and *Bombus pennsylvanicus*. However, since such empirical support is limited and because several studies have even failed to detect it in different systems (e.g. [7]–[10], [43], [44]), this trade-off may be less trivial and common than initially assumed.

*C. lineosa's* inferiority in the loess soil environment, where it is supposedly a habitat specialist, can be clarified through several non-mutually exclusive explanations. First, although *M. hyalinus* can be found in a variety of soils, including in hyper-arid regions characterized by extremely high temperatures [20], it is restricted to shaded micro-habitats (i.e., under trees or bushes), minimizing its exposure to these high temperatures. *C. lineosa*, in contrast, is usually found in micro-habitats exposed to direct sun, and the soil surface in such places may reach extremely high temperatures during the summer. Therefore, it is possible that *C. lineosa* has adapted to function at extremely high temperatures in addition to being a light soil specialist. Second, *C. lineosa* may compensate for its poor performance by reducing its metabolic rate to better resist starvation periods. Such a trade-off between intense foraging activity and the loss of body mass during starvation has already been shown in antlions [45]. Third, the relatively small differences in foraging efficiency between the two species in the loess (e.g., capture success of *M. hyalinus* was ∼16% higher than that of *C. lineosa*, irrespective of the prey ant species provided) may have little actual significance under stochastic natural conditions. Fourth, it is possible that *C. lineosa* inhabits light soils because its eggs or pupae better persist in these habitats. Alternatively, *C. lineosa* superiority may be evident only when considering other factors, such as growth rate and predation risk characterizing the different habitats. Clearly, these factors cannot be evident in short term behavioral experiments. Moreover, the role of predation in shaping the behavior of trap-building predators in general and pit building antlions in particular is still unclear (reviewed in [13], [46]). Finally, it is possible that the deeper pits of *C. lineosa* in the loess enable it to capture specific prey items not tested for in this experiment, which is an unlikely explanation, as antlions usually feed on ants (∼70% of their diet; [14]), and we have used different-sized ants from different locations, which are probably included in both species' diets.

Our prey capture success experiment clearly demonstrates that the success of both antlions is consistent among different prey ant species encountered, indicating that these opportunistic predators are diet generalists, while also suggesting that their spatial distribution should not be affected by the distribution of these ants. Obviously, prey capture success rates may change under natural conditions, as there are some ant species which help nest mates [47]. For example, Czechowski et. al. [48], observed that workers of *Formica sanguinea* caught by a larva of an antlion *Myrmeleon formicarius* can induce rescue behavior in their nest mates. Typical rescue behavior involves both the attempts to pull away the attacked ant by tugging at its limbs, and rapid, intense digging behavior. Such nest mate behavior, which can reduce the prey capture success of both antlion species, could not have been detected in our experiments, as each antlion received only one prey item. However, the fact that the response time to prey of *C. lineosa* is slower than that of *M. hyalinus*, strongly suggests that such rescue behavior may reduce the prey capture success of the former (i.e., habitat specialist) to a higher extent.

According to the theory of habitat selection, animals should select the habitat in which their fitness is higher [49]. Our habitat selection experiment indicates that when alone, each antlion species prefers the soil type providing higher prey capture success and characterizing its primary habitat-of-origin (Fig. 7). Surprisingly, although discrimination of the preferred habitat is critical, especially for *C. lineosa* due to its reduced ability to capture prey in the sandy soil, it appears, from our habitat selection experiment, that this species is significantly less selective compared to *M. hyalinus*. One possible explanation is that female *C. lineosa* oviposit their eggs in light soils far away from the alternative sandy soils, so that the larvae's probability of encountering a different soil type is relatively low. In other words, *C. lineosa* larvae less frequently exercise such discrimination between soils, and thus are more likely to make mistakes in choosing the correct habitat. Habitat selection practiced by the ovipositing females can greatly influence the future success of their progenies. Several studies have suggested that habitat selection in pit-building antlions is largely determined by the ovipositing female [50], [51]. Nevertheless, this study demonstrates that larvae of both species are capable of correcting their mother's choice by relocating and selecting the habitat which maximizes their prey capture success. Active habitat selection of antlion larvae, although relatively limited in scale, has been shown among microhabitats of different substrates [12], [17] and of different illumination levels [21], [36].

Specialization for light soils such as loess is not trivial, especially in arid and semi-arid environments where the above-ground net productivity of this soil type is much lower than that of coarse-textured sandy soils (i.e., the inverse texture effect; [52], [53]). Increased productivity is expected to correlate with increased potential prey biodiversity and abundance (e.g. [54]). We thus suggest that some mechanism compensates for this reduced insect abundance, such as low inter-specific competition. To summarize, the broad habitat niche breadth characterizing *M. hyalinus* may explain why its abundance, over large geographical scales, is higher than that of *C. lineosa*, which utilizes a narrower habitat range (i.e., being limited to light soil habitats). Since both antlions are opportunistic predators, their spatial distribution should be less affected by prey community structure. Finally, we suggest that habitat specialization in *C. lineosa* is either an evolutionary dead end [55], or, more likely, that this species' superiority in light soils may only be evident when considering additional niche axes such as starvation endurance and thermal conditions. In a broader context, we suggest that specialization should be examined while considering the multidimensional nature of the ecological niche.
